# Epidural esketamine and morphine for postoperative analgesia after caesarean delivery: A pilot study

**DOI:** 10.3389/fsurg.2022.988392

**Published:** 2023-01-06

**Authors:** Ju Tang, Zhiguo Zheng, Qijun Ran, Feng Zhao, Yao Wang, Feng Hu, Chao Yang, Xiaoyong Tan

**Affiliations:** ^1^ Department of Anesthesiology, People’s Hospital of Xuanhan County, Dazhou, China; ^2^ Department of Anesthesiology, The First Medical Center of PLA General Hospital, Beijing, China; ^3^ Department of Science and Education Section, People’s Hospital of Xuanhan County, Dazhou, China

**Keywords:** esketamine, morphine, cesarean section, analgesia, pain

## Abstract

**Objective:**

The aim of this study was to determine whether the addition of esketamine to morphine would improve postoperative analgesia after cesarean section.

**Methods:**

Parturients who planned for a cesarean delivery using combined spinal–epidural anesthesia with a request for postoperative anesthesia were randomly divided into four groups (A, B, C, and D). When the surgery was completed, the parturients in groups A, B, C, and D were administered 2 mg morphine, 0.25 mg/kg of esketamine, 0.25 mg/kg of esketamine plus 2 mg morphine hydrochloride, and 0.25 mg/kg of esketamine plus 1 mg morphine through the epidural catheters, respectively. The postoperative pain at rest, pain with movement, the number of rescue analgesics, and adverse effects were evaluated for 48 h after cesarean delivery.

**Results:**

A total of 119 parturients were included in this study, including 30 cases in group A, 30 cases in group B, 30 cases in group C, and 29 cases in group D. All visual analog scale (VAS) scores at rest and with movement were much lower in group C as compared with those in group A and group B (*P *< 0.05). Moreover, VAS scores at rest were also lower in Group C than in group D for 24 h (*P *< 0.05). Corresponding to the low pain scores, parturients in group C also required less rescue analgesia as compared with the other three groups (*P* = 0.021 for C vs. A, *P* < 0.001 for C vs. B, and *P* < 0.001 for C vs. D). There were no statistically significant differences between the four study groups with regard to the incidence of adverse events (*P *> 0.05).

**Conclusions:**

The addition of esketamine to morphine improved postoperative analgesia after cesarean section without increasing the incidence of adverse events.

## Introduction

Cesarean section is the most common surgery in the obstetrics department, accounting for 25%–45% of all births in China (1, 2). Post-cesarean pain control is still a challenge for the postoperative nursing of parturients. Pain after cesarean section can result in a delay in the recovery of parturients and their ability to return to daily functional activities (3), increasing the risk of thromboembolic events and postpartum depression (4, 5). Furthermore, post-cesarean pain can delay the breastfeeding of newborns, affecting the intimate communication between mothers and their infants (6).

Satisfactory post-cesarean pain management has been reported to be associated with a shorter hospital stay, improved mobility, and greater satisfaction in parturients (7). Several pain reduction protocols have been suggested for parturients after cesarean section. Among them, morphine and its derivatives are the most widely used (8). Morphine exerts a rapid analgesic effect and lasts for a long time. Its effect in relieving post-cesarean pain is well established (9, 10). However, the side effects may limit the use of morphine after cesarean section.

Many non-opioid analgesics have been used in combination with morphine to maintain analgesic efficacy, reduce postoperative use of opioids, and decrease opioid-related adverse effects (11). Esketamine, a nonselective N-methyl-D-aspartic acid (NMDA) receptor inhibitor, possesses non-opioid analgesic properties. Esketamine is commonly used for the treatment of resistant depression (6). In recent years, the analgesic effect of esketamine has drawn increasing attention. Lei et al. (12) found that esketamine effectively reduced acute and chronic pain after thoracoscopy and pulmonary surgery under general anesthesia. Nielsen et al. (13) reported that intraoperative esketamine reduced postoperative pain and opioid use after spine surgery. A study conducted by Wang et al. (6) confirmed that esketamine not only reduced pain but also decreased the incidence of postpartum depression in parturients who underwent cesarean section. However, no study has been performed to determine the effects of esketamine combined with morphine on pain control after cesarean section.

Thus, this single-center prospective study was conducted to determine whether the addition of esketamine to morphine would improve postoperative analgesia after cesarean section.

## Subjects and methods

### Subjects

The inclusion criteria were as follows: (1) nulliparous parturients who were scheduled for elective cesarean delivery under spinal–epidural anesthesia; (2) parturients who had requested postoperative analgesia; (3) parturients aged between 20 and 35 years old; (4) parturients who had a full-term pregnancy; (5) parturients who were identified as having a singleton pregnancy; and (6) parturients who were categorized as having an American Society of Anaesthesiologists (ASA) physical status of 1 or 2. This study has been registered, ClinicalTrials.gov Identifier: NCT05582135.

The key exclusion criteria were as follows: (1) parturients with severe internal, surgical, or obstetric comorbidities (including spinal deformities, hypertension, placental abruption, cholestasis in pregnancy, asthma, heart disease, and abnormal coagulation parameters); (2) parturients with a known allergy to the drugs used in this study; (3) parturients with severe mental illness who could not comply with doctors’ instructions; and (4) parturients with chronic pain syndrome, which is defined as pain that persists for a period longer than 3 months (14).

To ensure allocation concealment, the allocation sequence was randomly generated by computer by someone independent of the trial. The parturients enrolled were randomly divided by a computer-generated simple randomization schedule into four groups (A, B, C, and D) in a 1:1:1:1 ratio. The analgesic drugs were prepared by the nurse according to the random allocation and numbered accordingly. Enrolled parturients were given the corresponding numbered analgesic drugs. Neither the trial participants nor researchers were aware of the treatment allocation after randomization.

### Procedure

During the preoperative visit, the parturients were taught how to use the visual analog scale (VAS) for assessing pain. After entering the operation room, the standard anesthesia monitors were used for the parturients, including electrocardiogram, noninvasive blood pressure (BP), heart rate (HR), and peripheral oxygen saturation (SPO_2_). Spinal anesthesia was performed in the left lateral position at the L3–4 level using the needle-through-needle technique. Then, 2 ml of bupivacaine (5 mg/ml) was injected into the subarachnoid space, and an epidural catheter was inserted cephalad 3 cm rapidly. The parturients were then placed in the supine position, and surgery was started when the T6 sensory block was achieved.

The analgesic drugs were given to the parturients immediately upon completion of surgery. The parturients in group A were administered 2 mg morphine hydrochloride through the epidural catheter. The parturients in group B were administered 0.25 mg/kg of esketamine. The parturients in group C were administered 0.25 mg/kg of esketamine in combination with 2 mg morphine hydrochloride. The parturients in group D were administered 0.25 mg/kg of esketamine in combination with 1 mg morphine hydrochloride. Sterile saline was added to all combinations of the analgesic drugs to make a total volume of 8 ml. If the parturients still complained of pain after analgesic drug administration, rescue analgesia was administered by intramuscular injection of 100 mg tramadol, which was repeated if necessary.

### Outcome measures

The pain scores at rest and on movement were evaluated at 2, 4, 8, 12, 24, and 48 h after cesarean delivery by the parturients themselves using a VAS based on a linear scale from 0 to 10, where 0 represented an absence of pain and 10 represented maximal pain. The number of rescue analgesics required within 48 h of surgery was also recorded and compared. For safety, the systolic blood pressure (SBP), diastolic blood pressure (DBP), HR, and SpO_2_ were recorded at 2, 4, 8, 12, 24, and 48 h after drug administration, postoperatively. Adverse events that occurred after the administration of study drugs were also recorded.

### Statistical analysis

Based on previous data (7), we assumed that the average VAS score at rest at 24 h after cesarean delivery in the combination groups (group C and D) would be 1 point lower than that in the morphine hydrochloride alone (group A) group and that the average VAS score at 24 h after cesarean delivery in the esketamine alone (group B) group would be similar to group A. To detect a 1-point difference in the 0-to-10 VAS score, assuming a 1.15 SD (7), a sample size of 96 women (24 in each group) was calculated to suffice, with a power of 95% and an alpha error of 0.05. Considering that the randomized subjects might be lost for follow-up for various reasons, approximately 120 subjects were suggested to be included in this study. The sample size was processed using PASS 15.0 software.

The data in this study were analyzed using the IBM SPSS Statistics for Windows, Version 22.0 (Armonk, NY, United States) statistical package. Quantitative data were described as mean ± SD or median with 95% confidence interval. Categorical data were described as numbers and percentages. The Kolmogorov–Smirnov test was used to determine whether the quantitative data followed a normal distribution. The Chi-square test or Fisher's exact test was used for comparison of categorical data. One-way analysis of variance (ANOVA) was used for comparison of quantitative data. Repeated measures such as the VAS pain scores were assessed by the repeated measures ANOVA. The *post hoc* Bonferroni correction was done for multiple comparisons. A *P*-value <0.05 was considered as statistically significant.

## Results

**Figure 1:**
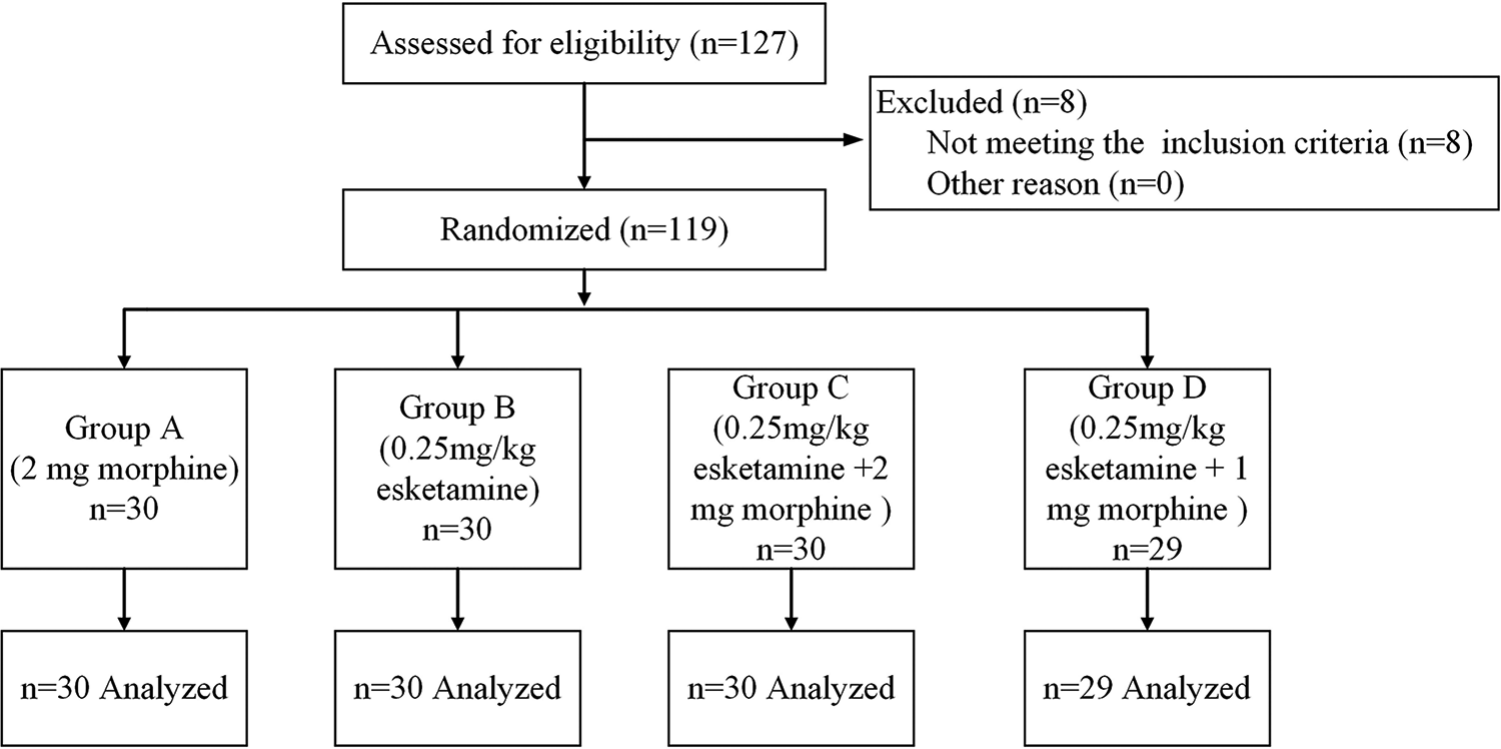
Flow diagram of all patients included in the four study groups.

From July 2020 to December 2021, 127 parturients were assessed for enrollment in this study. Eight were excluded before randomization based on the exclusion criteria (two parturients with a known allergy to esketamine, three with chronic pain syndrome, and three with severe internal comorbidities). A total of 119 parturients were finally included in this study, including 30 cases in group A, 30 cases in group B, 30 cases in group C, and 29 cases in group D (Figure 1). The baseline characteristics, including age, height, body weight, body mass index, duration of surgery, gestational age, and ASA physical status, were comparable among the four groups (Table 1).

**Table 1:** The characteristics of the parturients enrolled in this study.

Characteristic	Group A (*n* = 30)	Group B (*n* = 30)	Group C (*n* = 30)	Group D (*n* = 29)	*P*-value
Age (years, mean ± SD)	27.67 ± 3.63	27.83 ± 4.11	28.20 ± 4.06	26.66 ± 3.69	0.469
Height (cm, mean ± SD)	157.80 ± 4.05	156.33 ± 6.00	156.23 ± 5.92	156.31 ± 5.47	0.628
Weight (kg, mean ± SD)	67.23 ± 8.23	67.87 ± 8.78	65.47 ± 8.26	68.97 ± 6.55	0.402
BMI (kg/m^2^, mean ± SD)	27.01 ± 2.55	27.70 ± 2.70	26.78 ± 2.70	28.23 ± 2.29	0.127
Duration of surgery (min, mean ± SD)	52.87 ± 11.77	51.43 ± 10.25	51.17 ± 13.91	49.34 ± 10.79	0.722
Gestational age (weeks, mean ± SD)	38.92 ± 1.24	38.56 ± 1.13	38.37 ± 1.20	38.52 ± 1.44	0.197
ASA classification *n* (%)					0.667
Class I	21 (70.00)	18 (60.00)	22 (73.33)	21 (72.41)	
Class II	9 (30.00)	12 (40.00)	8 (26.67)	8 (27.59)	

BMI, Body mass index; ASA, American Society of Anesthesiologists.

Group A: administrated with 2 mg morphine hydrochloride; group B: administrated with 0.25 mg/kg of esketamine; group C: administrated with 0.25 mg/kg of esketamine in combination with 2 mg morphine hydrochloride; and group D: administrated with 0.25 mg/kg of esketamine in combination with 1 mg morphine hydrochloride.

Postoperative VAS scores for pain at rest are shown in Table 2. VAS scores were much lower for group C compared with group A or B for all the time points (*P* < 0.05). Moreover, group C had lower VAS scores compared to group D at all the time points except for at 24 h (*P* = 0.103) and 48 h (*P* = 0.098). There was no significant difference between groups A and D for the VAS scores at rest and at all the time points. However, group D had lower VAS scores when compared with group B at the time points of 2, 4, 8, and 12 h (*P* < 0.05).

**Table 2:** Postoperative pain score at rest in study groups.

Time points	Group A (*n* = 30)	Group B (*n* = 30)	Group C (*n* = 30)	Group D (*n* = 29)	*P*-value
2 h	2.20 ± 1.16	2.63 ± 1.27	0.60 ± 0.77	1.62 ± 1.18	<0.001
*P*-value^b^	P_AB _= 0.133, P_AC_ <_ _0.001, P_AD_ = 0.047, P_BC_ < 0.001, P_BD _= 0.001, P_CD_ = 0.001				
4 h	3.63 ± 1.13	5.07 ± 1.11	2.13 ± 1.01	3.31 ± 1.14	<0.001
*P*-value^b^	P_AB _< 0.001, P_AC_ <_ _0.001, P_AD_ = 0.261, P_BC_ < 0.001, P_BD _< 0.001, P_CD_ < 0.001				
8 h	4.80 ± 1.06	6.00 ± 1.26	3.20 ± 1.16	4.48 ± 1.15	<0.001
*P*-value^b^	P_AB _< 0.001, P_AC_ <_ _0.001, P_AD_ = 0.296, P_BC_ < 0.001, P_BD _< 0.001, P_CD_ < 0.001				
12 h	4.90 ± 1.40	5.13 ± 1.01	3.37 ± 1.10	4.48 ± 1.21	<0.001
*P*-value^b^	P_AB _= 0.448, P_AC_ <_ _0.001, P_AD_ = 0.180, P_BC_ < 0.001, P_BD _= 0.038, P_CD_ < 0.001				
24 h	3.70 ± 2.09	4.13 ± 1.22	2.73 ± 1.05	3.38 ± 1.47	0.005
*P*-value^b^	P_AB _= 0.269, P_AC_ =_ _0.015, P_AD_ = 0.417, P_BC_ < 0.001, P_BD _= 0.058, P_CD_ = 0.103				
48 h	1.80 ± 1.32	2.03 ± 1.19	1.03 ± 0.99	1.55 ± 1.24	0.011
*P*-value^b^	P_AB _= 0.451, P_AC_ =_ _0.014, P_AD_ = 0.426, P_BC_ = 0.002, P_BD _= 0.124, P_CD_ = 0.098				
Repeated ANOVA^a^	<0.001	<0.001	<0.001	<0.001	

Group A (administrated with 2 mg morphine hydrochloride), Group B (administrated with 0.25 mg/kg of esketamine), Group C (administrated with 0.25 mg/kg of esketamine in combination with 2 mg morphine hydrochloride and Group D (administrated with 0.25 mg/kg of esketamine in combination with 1 mg morphine hydrochloride).

^a^ Repeated measure ANOVA test was used to analyze the effect of different procedures over time.

^b^ Post hoc comparisons with Bonferroni corrections. P_AB _= A vs. B, P_AC_ = A vs. C, P_AD_ = A vs. D, P_BC_ = B vs. C, P_BD _= B vs. D, P_CD_ = C vs. D.

Postoperative VAS scores for pain with movement are shown in Table 3. From the time point of 4 h, VAS scores were much lower for group C compared with groups A or B (*P* < 0.05). Furthermore, at the time points of 8, 12, and 24 h, group C had lower VAS values compared to group D (*P* < 0.05). There was no significant difference between groups A and D regarding the VAS scores with movement at all the time points. When compared with group B, group D only had significantly lower VAS scores at the time points of 4 and 8 h (*P* = 0.003 and *P* < 0.001, respectively).

**Table 3:** Postoperative pain score with movement in study groups.

Time points	Group A (*n* = 30)	Group B (*n* = 30)	Group C (*n* = 30)	Group D (*n* = 29)	*P*-value
2 h	0.00 ± 0.00	0.41 ± 1.64	0.00 ± 0.00	0.00 ± 0.00	0.134
*P*-value^b^	P_AB _= 0.053, P_AC_ = 1.000, P_AD_ = 1.000, P_BC_ = 0.053, P_BD _= 0.055, P_CD_ = 1.000				
4 h	2.17 ± 2.95	4.31 ± 3.78	0.83 ± 1.76	2.00 ± 2.95	<0.001
*P*-value^b^	P_AB _= 0.006, P_AC_ =_ _0.082, P_AD_ = 0.828, P_BC_ < 0.001, P_BD _= 0.003, P_CD_ = 0.131				
8 h	6.87 ± 1.07	8.17 ± 1.07	5.10 ± 1.67	6.45 ± 1.92	<0.001
*P*-value^b^	P_AB _= 0.001, P_AC_ <_ _0.001, P_AD_ = 0.280, P_BC_ < 0.001, P_BD _< 0.001, P_CD_ = 0.001				
12 h	7.00 ± 1.51	7.31 ± 1.04	5.57 ± 1.43	6.93 ± 1.16	<0.001
*P*-value^b^	P_AB _= 0.362, P_AC_ <_ _0.001, P_AD_ = 0.839, P_BC_ < 0.001, P_BD _= 0.270, P_CD_ < 0.001				
24 h	5.73 ± 1.14	6.17 ± 0.69	4.97 ± 1.19	5.72 ± 1.13	0.001
*P*-value^b^	P_AB _= 0.132, P_AC_ =_ _0.009, P_AD_ = 0.975, P_BC_ < 0.001, P_BD _= 0.127, P_CD_ = 0.010				
48 h	4.13 ± 1.11	4.31 ± 0.81	3.53 ± 1.00	4.00 ± 1.10	0.026
*P*-value^b^	P_AB _= 0.504, P_AC_ =_ _0.024, P_AD_ = 0.614, P_BC_ = 0.004, P_BD _= 0.246, P_CD_ = 0.080				
Repeated ANOVA^a^	<0.001	<0.001	<0.001	<0.001	

Group A (administrated with 2 mg morphine hydrochloride), Group B (administrated with 0.25 mg/kg of esketamine), Group C (administrated with 0.25 mg/kg of esketamine in combination with 2 mg morphine hydrochloride and Group D (administrated with 0.25 mg/kg of esketamine in combination with 1 mg morphine hydrochloride).

^a^ Repeated measure ANOVA test was used to analyze the effect of different procedures over time.

^b^ Post hoc comparisons with Bonferroni corrections. P_AB _= A vs. B, P_AC_ = A vs. C, P_AD_ = A vs. D, P_BC_ = B vs. C, P_BD _= B vs. D, P_CD_ = C vs. D.

**Figure 2:**
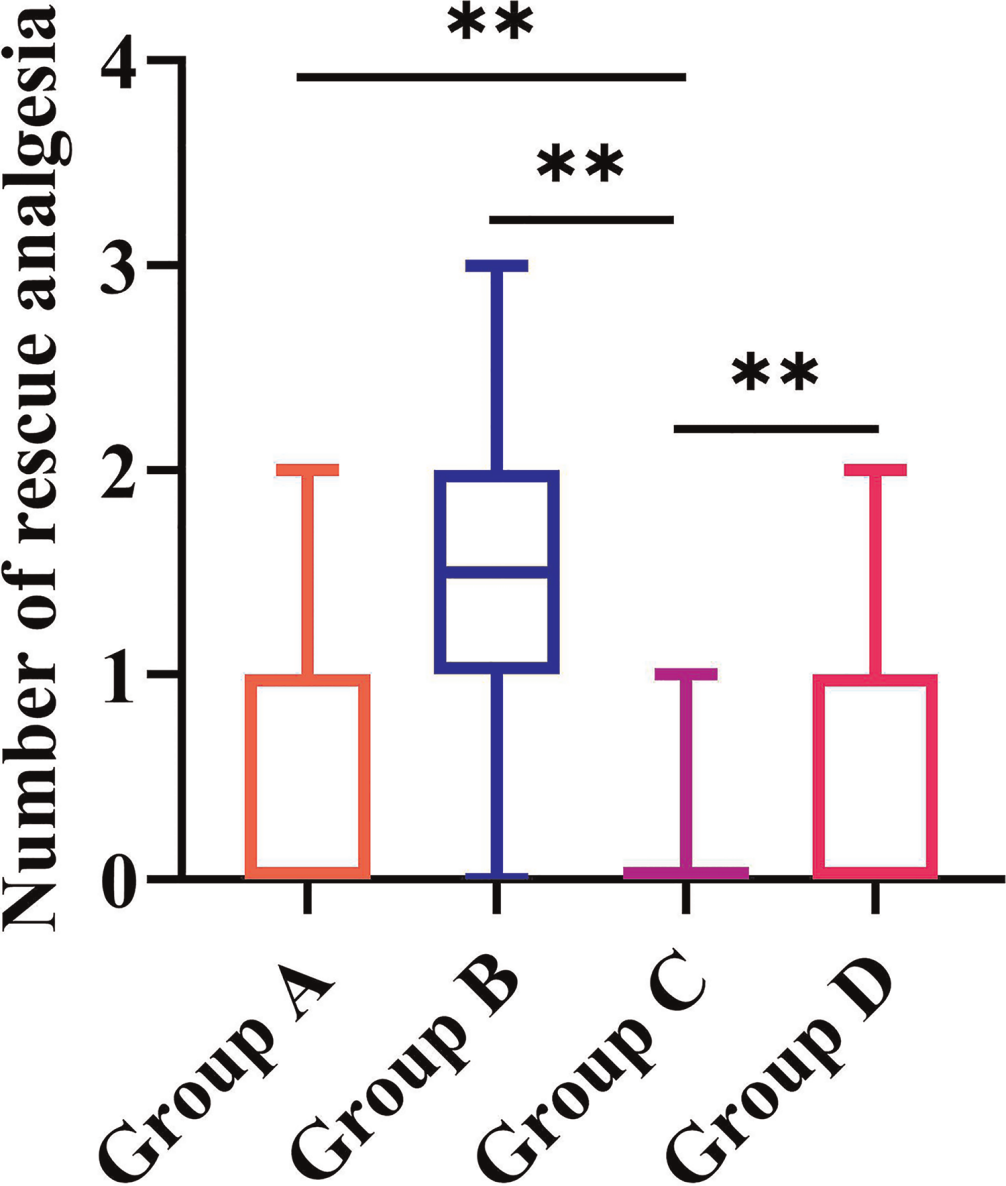
The results for rescue analgesia in the four study groups. Group A: administrated with 2 mg morphine hydrochloride; group B: administrated with 0.25 mg/kg of esketamine; group C: administrated with 0.25 mg/kg of esketamine in combination with 2 mg morphine hydrochloride; and group D: administrated with 0.25 mg/kg of esketamine in combination with 1 mg morphine hydrochloride. Data are presented as median (interquartile range). **P < 0.05.

The results for rescue analgesics are shown in Figure 2. Compared with groups A, B, and D, group C required less rescue analgesia within the first 48 h after surgery (*P* = 0.021 for C vs. A, *P* < 0.001 for C vs. B, and *P* < 0.001 for C vs. D, respectively).

**Figure 3:**
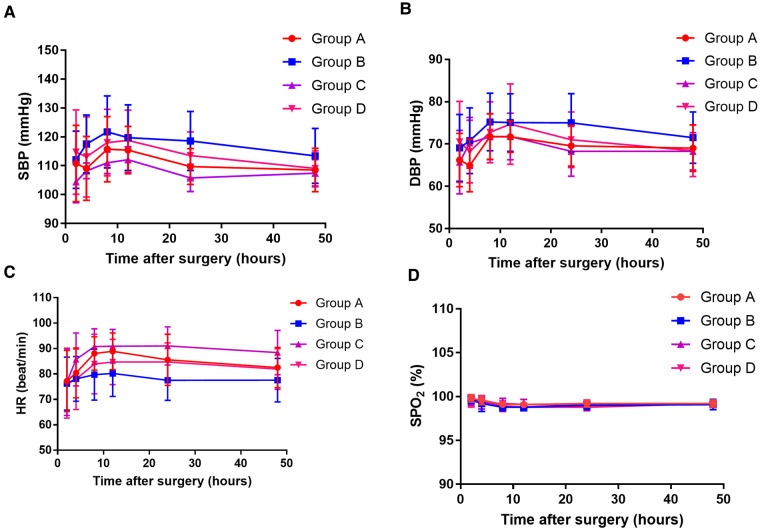
The systolic blood pressure (SBP) value (**A**), diastolic blood pressure (DBP) value (**B**), heart rate (HR) value (**C**), and oxygen saturation (SPO_2_) value (**D**) at 10, 20, 30, 40, and 50 h after surgery among the four groups. Group A: administrated with 2 mg morphine hydrochloride; group B: administrated with 0.25 mg/kg of esketamine; group C: administrated with 0.25 mg/kg of esketamine in combination with 2 mg morphine hydrochloride; and group D: administrated with 0.25 mg/kg of esketamine in combination with 1 mg morphine hydrochloride.

There were no significant differences in the SBP, DBP, HR, and SpO_2_ among the four groups at the six time points after surgery (two-way ANOVA, overall *P* = 0.099, 0.354, 0.105, and 0.476, respectively, Figure 3). No serious complications occurred during the study period. Nausea and/or vomiting occurred in five patients in group A, three in group B, four in group C, and three in group D. Pruritus occurred in four patients in group A, two in group B, two in group C, and one in group D. Dizziness occurred in one patient in group A, one in group B, two in group C, and three in group D. There was no statistically significant difference between the four groups regarding the incidence of adverse events (Table 4).

**Table 4:** Summary of adverse events in study groups.

	Group A (*n* = 30)	Group B (*n* = 30)	Group C (*n* = 30)	Group D (*n* = 29)	*P*-value
Nausea/vomiting, *n* (%)	5 (16.67)	3 (10.00)	4 (13.33)	3 (10.34)	0.929
Pruritus, *n* (%)	4 (13.33)	2 (6.67)	2 (6.67)	1 (3.45)	0.625
Dizzy, *n* (%)	1 (3.33)	1 (3.33)	2 (6.67)	3 (10.34)	0.590
Hypertension, *n*	1	0	0	1	—
Hypotension, *n*	0	1	1	0	—
Bradycardia, *n*	1	1	1	0	—
Tachycardia, *n*	0	1	0	0	—
Respiratory depression, *n*	0	0	0	0	—

Group A: administrated with 2 mg morphine hydrochloride; group B: administrated with 0.25 mg/kg of esketamine; group C: administrated with 0.25 mg/kg of esketamine in combination with 2 mg morphine hydrochloride; and group D: administrated with 0.25 mg/kg of esketamine in combination with 1 mg morphine hydrochloride.

## Discussion

In the present study, we found that the analgesic regime of epidural esketamine and morphine loaded immediately after cesarean delivery could significantly relieve postoperative pain and reduce the requirement of rescue analgesia compared with morphine or esketamine alone. Furthermore, this combination was not associated with an increased incidence of adverse events.

As an NMDA-receptor antagonist, esketamine has been used effectively to treat depressive disorders (6). Furthermore, esketamine has been reported to be useful for preventing hyperalgesia and controlling pain after surgery (13, 15). Suppa et al. (16) reported that esketamine had an anti-hyperalgesic action that could effectively control postoperative pain in parturients who underwent cesarean delivery. Han et al. (17) found that low-dose esketamine used as an adjuvant in patient-controlled intravenous analgesia significantly reduced the incidence of postpartum depression and relieved pain after cesarean delivery. Similarly, Wang et al. (6) also confirmed that low-dose esketamine could reduce both post-cesarean pain and the incidence of postpartum depression in parturients.

Because of its ability to prevent hyperalgesia and central sensitization without respiratory depression, ketamine has been suggested as an adjuvant drug in opioid analgesia (18). Esketamine has a higher affinity for NMDA receptors than ketamine (6). It is also reported to have a shorter sedation time and fewer side effects than ketamine (19). Thus, esketamine has been used as an adjuvant drug in multimodal analgesia. The administration of esketamine in combination with opioids in the nonpregnant population has been described previously. Brinck et al. (20) found that patient-controlled opioid analgesia containing esketamine could decrease cumulative opioid consumption at 24 h after major lumbar spinal fusion surgery without additional adverse effects. Snijdelaar et al. (21) reported that pain scores and cumulative morphine consumption were significantly lower in patients who received esketamine/morphine compared to those who received morphine alone after radical prostatectomy. Lyu et al. (22) demonstrated that esketamine combined with sufentanil could provide satisfactory analgesia in patients who had undergone laparoscopic radical resection.

In the present study, esketamine in combination with morphine was demonstrated to be effective for parturients who underwent cesarean delivery. We found that epidural 0.25 mg/kg esketamine and 2 mg morphine immediately after delivery could significantly relieve postoperative pain at rest and with movement. Epidural anesthesia is one of the treatment protocols that have been suggested for women who undergo cesarean delivery (23). Previous studies reported that epidural morphine significantly prolongs analgesia after cesarean delivery (24). Here, we found that parturients who received epidural 0.25 mg/kg esketamine combined with 2 mg morphine had much lower pain scores for 48 h after cesarean delivery (including pain at rest and pain with movement) than those who received 2 mg morphine or 0.25 mg/kg esketamine alone. At most of the time points in the study period, parturients who received epidural 0.25 mg/kg esketamine combined with 2 mg morphine also had lower pain scores than those who received epidural 0.25 mg/kg esketamine and 1 mg morphine. Corresponding to the results of VAS scores, parturients in the group who received 0.25 mg/kg esketamine combined with 2 mg morphine required less rescue analgesia within the first 48 h postoperatively as compared with the other three groups. These results indicate that the addition of esketamine to morphine improves postoperative analgesia after cesarean section.

There was no statistically significant difference between the four study groups with regard to the incidence of adverse events. Additionally, there were no significant differences in the SBP, DBP, HR, and SpO_2_ among the four groups at all the six time points after surgery. Thus, the addition of esketamine to morphine does not increase the risk of adverse events.

Furthermore, in this study, it was also found that parturients who received epidural 0.25 mg/kg esketamine combined with 1 mg morphine had similar pain scores compared with those who received 2 mg morphine. Thus, the addition of esketamine to morphine may reduce the dose of morphine required and yet achieve the same effect. This result should be confirmed in further studies.

The present study has several limitations. First, this study was carried out in a single center with a small sample size. A multicenter study with a larger sample size is needed to confirm the conclusions of this study. Second, the assessment of post-cesarean pain was based on the VAS, which is completely subjective. More objective methods should be considered in further studies. Finally, previous studies have shown that esketamine reduces the incidence of postpartum depression in parturients (6). Due to the short study period and small sample size, we did not observe the effect of esketamine in reducing postpartum depression. This effect should be investigated in further studies.

## Conclusion

This study showed that epidural esketamine and morphine immediately after cesarean delivery significantly relieves postoperative pain and reduces the requirement of rescue analgesia, compared with using morphine or esketamine alone. Thus, the addition of esketamine to morphine improves postoperative analgesia after cesarean section without increasing the risk of adverse events.

## Data Availability

The original contributions presented in the study are included in the article/Supplementary Material, further inquiries can be directed to the corresponding authors.
